# Influence of pH on Inulin Conversion to 2,3-Butanediol by *Bacillus licheniformis* 24: A Gene Expression Assay

**DOI:** 10.3390/ijms241814065

**Published:** 2023-09-14

**Authors:** Lidia Tsigoriyna, Alexander Arsov, Emanoel Gergov, Penka Petrova, Kaloyan Petrov

**Affiliations:** 1 Institute of Chemical Engineering, Bulgarian Academy of Sciences, 1113 Sofia, Bulgaria; 2 Institute of Microbiology, Bulgarian Academy of Sciences, 1113 Sofia, Bulgariappetrova@microbio.bas.bg (P.P.)

**Keywords:** 2,3-Butanediol, *Bacillus licheniformis*, *sacB*, *sacC*, real-time RT-PCR

## Abstract

2,3-Butanediol (2,3-BD) is an alcohol highly demanded in the chemical, pharmaceutical, and food industries. Its microbial production, safe non-pathogenic producer strains, and suitable substrates have been avidly sought in recent years. The present study investigated 2,3-BD synthesis by the GRAS *Bacillus licheniformis* 24 using chicory inulin as a cheap and renewable substrate. The process appears to be pH-dependent. At pH 5.25, the synthesis of 2,3-BD was barely detectable due to the lack of inulin hydrolysis. At pH 6.25, 2,3-BD concentration reached 67.5 g/L with rapid hydrolysis of the substrate but was accompanied by exopolysaccharide (EPS) synthesis. Since inulin conversion by bacteria is a complex process and begins with its hydrolysis, the question of the acting enzymes arose. Genome mining revealed that several glycoside hydrolase (GH) enzymes from different CAZy families are involved. Five genes encoding such enzymes in *B. licheniformis* 24 were amplified and sequenced: *sacA*, *sacB*, *sacC*, *levB,* and *fruA*. Real-time RT-PCR experiments showed that the process of inulin hydrolysis is regulated at the level of gene expression, as four genes were significantly overexpressed at pH 6.25. In contrast, the expression of *levB* remained at the same level at the different pH values at all-time points. It was concluded that the *sacC* and *sacA/fruA* genes are crucial for inulin hydrolysis. They encode exoinulinase (EC 3.2.1.80) and sucrases (EC 3.2.1.26), respectively. The striking overexpression of *sacB* under these conditions led to increased synthesis of EPS; therefore, the simultaneous production of 2,3-BD and EPS cannot be avoided.

## 1. Introduction

The Green Deal or the new European strategy to make the continent climate-neutral by 2050 requires a circular economy and the valorization of renewable feedstock [1]. The related development of microbial biotechnologies needs new energy resources and substrates (such as plant biomass) to obtain chemical compounds in alternative ways, avoiding the use of fossil fuels. Such an example is the production of 2,3-butanediol (2,3-BD), with the most rapidly growing market [2], projected to reach USD 300 million by the end of 2030 [3]. It is used as a fuel additive, antifreeze, solvent, flavoring agent, platform chemical for polyesters, polyurethanes, and methacrylate synthesis, and for the production of rubber, drugs, ointments, and antiperspirants [4,5,6,7]. The possibility of the biotechnological synthesis of 2,3-BD dates back to 1906 when Harden and Walpole investigated its production by *Klebsiella pneumoniae* [6]. In 1926, Donker noticed it as a metabolic product of *Paenibacillus polymyxa* (*Bacillus polymixa*), but the idea of the industrial production of 2,3-BD was first proposed by Fulmer et al. in 1933 [4]. During World War II, interest in the production of 2,3-BD became more significant than ever because of its use as a precursor of synthetic rubber. The first pilot plant for the production of 2,3-BD was built, and the process was carried out via the microbial fermentation of the *K. oxytoca* and *P. polymyxa* strains. However, large-scale industrial production never took off as it turned out to be more cost-effective to produce 1,3-butadiene from petroleum [5]. In the 1970s, due to rising oil prices, interest in 2,3-BD biotechnology returned. Pilot plants were built again in the United States and later in China, this time with the idea of converting low-cost lignocellulosic wastes [4]. However, to date, despite great scientific and technological progress, industrial microbial production of 2,3-BD has not taken place.

**Figure 1:**
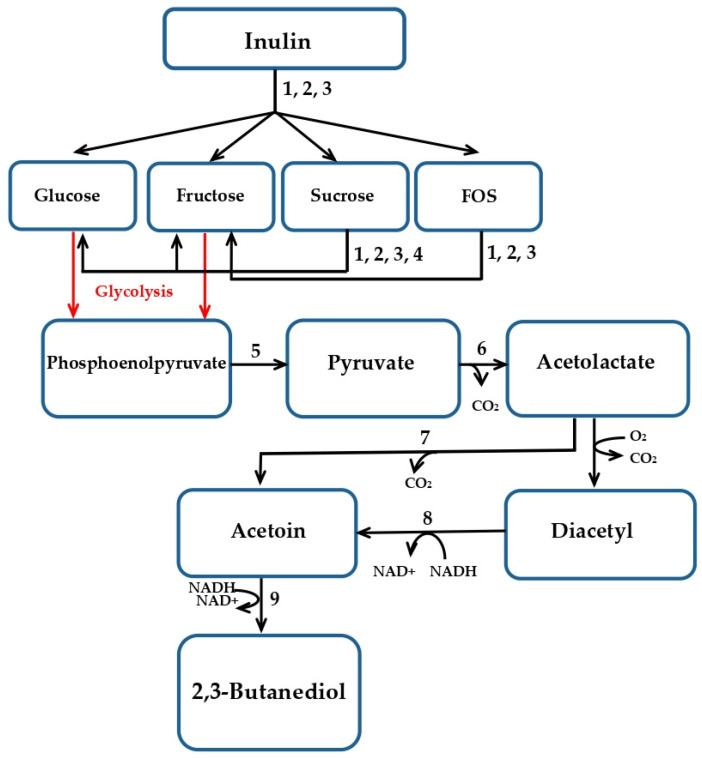
The metabolic pathway of inulin conversion to 2,3-butanediol by *B. licheniformis* (based on Maina et al. [6]. Designations: FOS, fructooligosaccharides; enzymes: 1, fructan β-fructosidase, EC 3.2.1.80; 2, endolevanase, EC 3.2.1.65; 3, β-fructofuranosidase, EC 3.2.1.26; 4, levansucrase, EC 2.4.1.10; 5, pyruvate kinase; 6, α-acetolactate synthase; 7, α-acetolactate decarboxylase; 8, diacetyl reductase (DAR); and 9, acetoin reductase (2,3-BD dehydrogenase). The conversion of acetolactate to diacetyl is spontaneous in the presence of oxygen.

The recent advance in microbial 2,3-BD production is marked by the very successful engagement of generally regarded as safe (GRAS) microorganisms belonging to the genera *Bacillus* [8,9,10,11] and *Paenibacillus* [12,13]. Among them, *B. licheniformis* stands out as the most promising 2,3-BD producer from glucose and fructose, reaching concentrations of up to 150 g/L and a yield close to the theoretical [14,15,16]. Moreover, *B. licheniformis* is also able to convert hydrolysates containing these two sugars into 2,3-BD, as *B. licheniformis* NCIMB 8059 utilizes starch hydrolysate [17], while *B. licheniformis* X10 uses corn stover hydrolysate [18]. Interest in converting inulin (the polysaccharide of D-fructose bonded with a β-(2→1) linkage and terminated with D-glucose) is even greater because it is the reserve carbohydrate of the Asteraceae family. These plants (chicory, Jerusalem artichoke, and dahlia) are C4 photosynthetic (fixing carbon dioxide into the four-carbon molecule of malate), able to grow on poor, infertile, and arid soils, and inappropriate for food crop cultivation [19]. The metabolic pathway of inulin conversion to 2,3-butanediol by *B. licheniformis* is presented in Figure 1.

In spite of inulin already having been applied as a substrate for microbial 2,3-BD production [20,21,22,23], its profitable use in simultaneous saccharification and fermentation (SSF) processes remains a subject of intense scientific research. Several attempts to increase the natural inulinase activity of *Bacillus* spp. strains via the cloning and expression of relevant genes have been performed. Li et al. [24] identified a levanase capable of inulin hydrolysis in *B. licheniformis* ATCC 14580^T^, cloned the responsible gene *sacC* in *E. coli*, and used the purified enzyme in an SSF process. However, the effective inulin conversion required several external additions of the purified enzyme (30 U/mL each).

**Figure 2:**
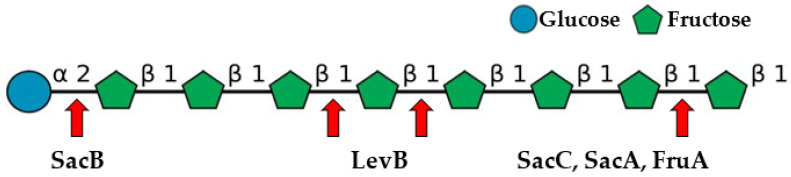
Scheme of the inulin molecule and the glycosidic bonds, which are the most usually attacked by different enzymes involved in inulin hydrolysis. Designations: α 2, α-(1→2) linkage; β 1, β-(2→1) linkage; SacA, β-fructofuranosidase, EC 3.2.1.26; SacB, levansucrase, EC 2.4.1.10; FruA, β-fructofuranosidase, EC 3.2.1.26; LevB, endolevanase, EC 3.2.1.65; and SacC, fructan β-fructosidase (exolevanase), EC 3.2.1.80. Inulin molecule was drawn using DrawGlycan-SNFG version 2.1 http://www.virtualglycome.org/DrawGlycan/ (accessed on 11 August 2023).

An overview of the literature reveals that inulin hydrolysis by *B. licheniformis* is a more complex process and cannot be attributed to the action (or regulation) of a single gene. Unraveling the genome of the type strain ATCC 14580^T^ showed the presence of several genes encoding glycoside hydrolase enzymes putatively related to inulin hydrolysis: *sacA*, *sacB*, *sacC*, *levB*, and *fruA*. Most of these enzymes have been individually characterized, such as sucrose-6-phosphate hydrolase (β-fructofuranosidase, invertase) [25], levansucrase [26,27,28,29], levanase SacC [24], and endolevanase LevB [30]. A scheme of the glycosidic bonds that these enzymes usually target is shown in Figure 2.

However, in the bacterial cell, the enzymes’ action or the regulation of their expression does not occur independently because glycoside hydrolases usually show cross-specificity to similar substrates. Furthermore, important characteristics of inulin-containing substrates are the length of the fructan chain (i.e., the degree of polymerization (DP)) and the remaining sugar content. The initial composition of the substrate depends on the plant source and the extraction technology and can predetermine the course of the whole fermentation process.

Our previous studies employed the excellent 2,3-BD producer *B. licheniformis* strain 24 [11,14,16,31]. Because the strain was able to achieve a record titer of 156.1 g/L of 2,3-BD from fructose [16] and displayed some natural inulinase activity [11], it was considered promising for inulin conversion to 2,3-BD as well. However, *B. licheniformis* 24 was capable of converting only certain types of inulin and was not able to produce 2,3-BD from Frutafit^®^ HD (highly dispersible, raw, and insoluble chicory flour), regardless of the cultivation conditions. The introduction of a heterologous inulinase gene (from *Lactobacillus*) into the strain resulted in an eightfold increase in its inulinase activity and full hydrolysis of 140 g/L of raw insoluble Frutafit^®^ HD inulin by the recombinant, but relatively low 2,3-BD and acetoin yields (18.5 g/L and 8.2 g/L, respectively) because of the accumulation of unconverted sucrose [31]. However, it was noticed that the strain was able to hydrolyze Frutafit^®^ CLR inulin (soluble chicory flour) under certain conditions. Thus, the present work aimed to investigate the mechanism of the inulin conversion of Frutafit^®^ CLR to 2,3-BD by elucidating the effects of different factors on the gene expression of the enzymes involved.

## 2. Results

### 2.1. Soluble and Insoluble Chicory Flour as a Substrate for 2,3-BD Production

**Figure 3:**
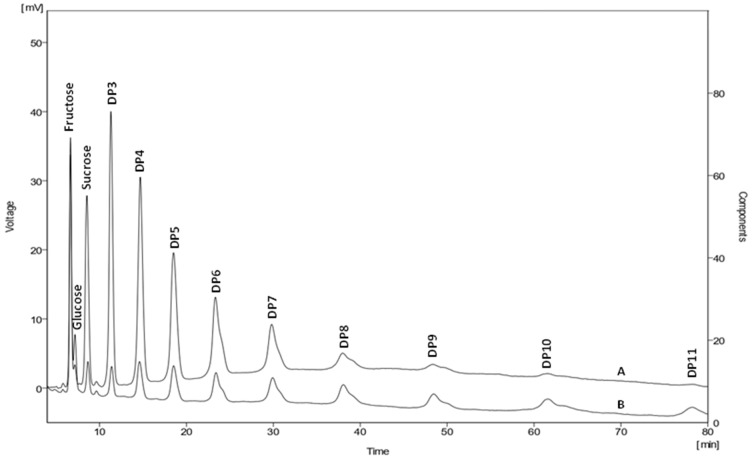
HPLC analysis of two different types of inulin-containing chicory flour after autoclaving at 120 °C for 20 min. Designations: (A) Sugar profile of Frutafit^®^ HD inulin (raw, insoluble chicory flour); (B) sugar profile of Frutafit^®^ CLR inulin (soluble chicory flour). Both inulin-containing substrates were analyzed as water solutions with a concentration of 100 g/L. DP, degree of polymerization.

To clarify the difference between Frutafit^®^ CLR and Frutafit^®^ HD, the contents of these substrates were compared using HPLC analysis after autoclaving their water solutions at 121 °C for 20 min (Figure 3). The results indicate that 100 g/L of the soluble chicory flour (Frutafit^®^ CLR) contained approximately 19.4 g/L of sugars (7.9 g/L of fructose, 1.5 g/L of glucose, and of 10 g/L sucrose), while insoluble Frutafit^®^ HD contained less than 10 g/L of mono- and disaccharides in total. Furthermore, the soluble Frutafit^®^ CLR contained oligosaccharides with a lower degree of polymerization, mainly DP3, DP4, and DP5 (Figure 3).

As *B. licheniformis* 24 was not able to convert Frutafit^®^ HD, all experiments described below were carried out using soluble chicory flour (Frutafit^®^ CLR) as a substrate.

### 2.2. Flask-Batch Fermentation of Inulin by B. licheniformis 24

**Figure 4:**
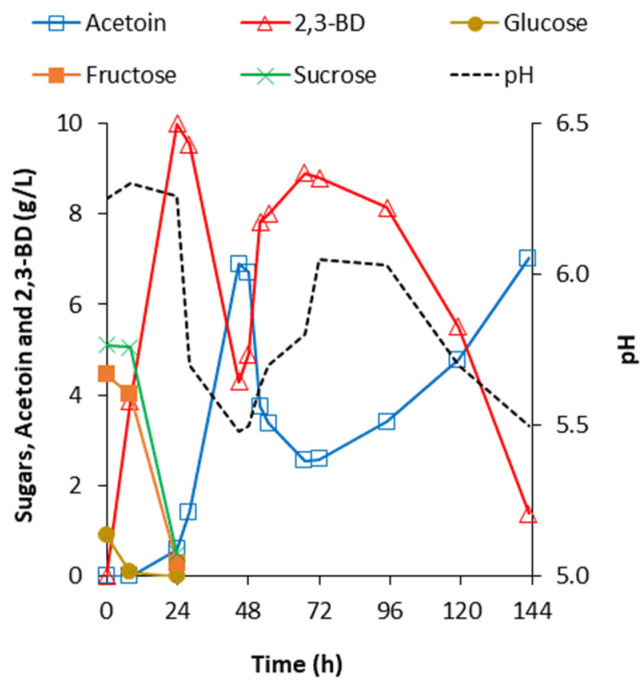
Utilization of 50 g/L of soluble chicory flour Frutafit^®^ CLR by *B. licheniformis* 24 in flask-batch fermentation in a rotary shaker at 37 °C and 200 rpm. The spontaneous pH fluctuation during the process is shown.

In flask-batch fermentation, when pH was not controlled, *B. licheniformis* 24 was formed 2,3-BD in two separate steps (Figure 4).

The first peak of 2,3-BD concentration was observed at the 24th h when the sugars were totally consumed. Then, 2,3-BD was converted to acetoin and the pH rapidly dropped from 6.2 to 5.5. After the 48th h, a new accumulation of 2,3-BD was observed as the pH rose to approximately 6.0. Finally, almost all formed 2,3-BD was converted to acetoin again, which was accompanied by a decrease in the pH to 5.5.

Obviously, the first accumulation of 2,3-BD was a result of sugar utilization, while the second was entirely a result of inulin conversion.

### 2.3. Effect of pH on Inulin Conversion to 2,3-BD

**Figure 5:**
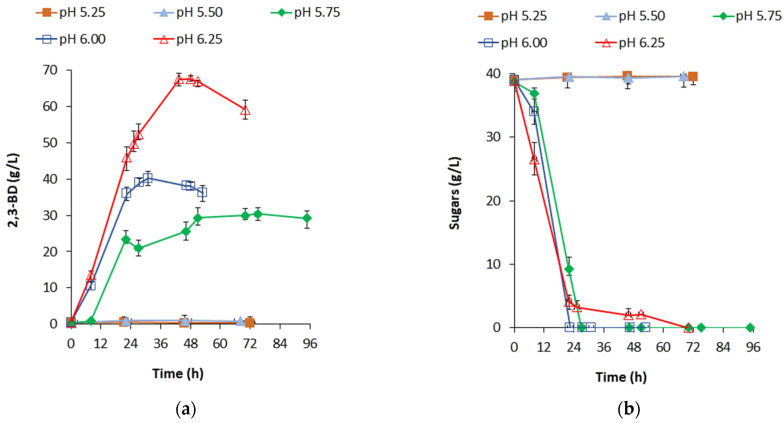
(**a**) Production of 2,3-butanediol by *B. licheniformis* from 200 g/L of soluble chicory flour at different pH values; (**b**) Sugar consumption by *B. licheniformis* in the fermentation of 200 g/L of the soluble chicory flour Frutafit^®^ CLR at different pH values. Fermentations were carried out in a bioreactor at 37 °C, with agitation at 500 rpm, and with aeration at 3.68 vvm (volume of air sparged per unit volume of growth medium per minute).

The influence of pH on 2,3-BD production was studied in the course of processes carried out in a fermenter and with an initial substrate concentration of 200 g/L of Frutafit^®^ CLR. At pH 6.25, the highest amount of 67.52 g/L of 2,3-BD was gained at approximately the 48th hour (Figure 5a). In this case, taking into account the overall sugar consumption rate (Figure 5b), simultaneous inulin hydrolysis and sugar consumption occurred. More than a third less was the maximum amount of 2,3-BD at pH 6.0 (40.20 g/L at the 31st h) and more than two times less at pH 5.75 (29.92 g/L) at approximately the 70th h. During the processes carried out at pH 5.25–pH 5.50, no 2,3-BD was produced, and no consumption of sugars occurred (Figure 5).

With the pH increase, the visual viscosity of the culture liquid increased correspondingly, suggesting the synthesis of EPS. At pH 5.75, and especially at pH 6.25, the most significant EPS production was observed. However, EPS synthesis was expected, since in our previous study conducted under the same fermentation conditions, *B. licheniformis* 24 formed EPS from monosaccharides, reaching yields of 9.64 g/L (from glucose) and 6.29 g/L (from fructose) [32].

### 2.4. Sequencing of GH Genes Involved in Inulin Hydrolysis by B. licheniformis 24

The elucidation of the gene set involved in inulin hydrolysis by *B. licheniformis* was performed by surveying the genomic database of the type strain ATCC 14580^T^ (2004, (NCBI GenBank acc. no. CP034569.1, BioProject PRJNA509976, Rey et al. [33]) based on information provided by KEGG (Kyoto Encyclopedia of Genes and Genomes). 

Separately, the sequences of the five genes potentially involved in inulin hydrolysis by *B. licheniformis* 24 were PCR amplified and sequenced, and the sequences were compared with the corresponding genes of the type strain. The sequences were deposited in the NCBI GeneBank under the accession numbers listed in Table 1.

Four of the respective enzymes belong to the GH32 family of glycosidases that hydrolyze O-glycosyl bonds, and one, a levansucrase, to the GH68 family of hexosyltransferases. Compared with the respective genes of the type strain *B. licheniformis* ATCC 14580^T^, the nucleotide sequences of *sacA*, *sacB*, and *sacC* of *B. licheniformis* 24 were identical. *levB* (99.79%) and *fruA* (99.12%) were highly similar but not identical. Several amino acid substitutions in the respective proteins were observed: phenylalanine was substituted with isoleucine at position 273 in LevB of *B. licheniformis* 24, and phenylalanine was changed to serine at position 220, and glutamate-256 to histidine in FruA. However, all amino acid substitutions were outside the catalytic triad (Glu-37, Glu-161, and Asp-215) and most probably did not affect the activities of both enzymes.

### 2.5. Real-Time Reverse Transcription PCR (RT-PCR)

Real-time RT-PCR investigation showed that four of the five genes implicated in the hydrolysis of inulin significantly altered their expression levels at different pH values (Table 2).

At pH 5.50, the five studied genes did not change their expressions compared with at pH 5.25. A large difference in gene expression was observed at pH 5.75. The expression of sucrases genes *fruA*/*sacA* increased 8–10-fold, the expression of *sacB* more than 12-fold, and that of *sacC* more than 23-fold relative to pH 5.25. In contrast, the expression of *levB* remained at the same level at the different pH values at all-time points.

At pH 6.25, at the 24th hour, the overexpression of four of the studied genes became strikingly high: *sacA*, *sacB*, *sacC*, and *fruA* expression levels raised 54 times (*fruA*), 66 times (*sacA*), 163 times (*sacC*), and 197 times (*sacB*). At the 48th h, a sharp drop to the baseline expression level for each respective gene occurred.

## 3. Discussion

Analyzing the current progress in 2,3-BD production from inulin, it is evident that a more detailed study of the hydrolysis processes of inulin and oligofructose by *B. licheniformis* is needed. The substrates containing inulin are quite diverse, for example, Jerusalem artichoke, which was used as a powder in [34], and hydrolysate or “extract”, with very limited information about its oligosaccharide structure [20,21,22,23,24]. Chicory flour of different manufacturers contains inulin with different DP and solubility, for instance, Orafti^®^ GR (92% inulin) is soluble in a concentration of 12 g/L, while Orafti^®^ HPX is in only 5 g/L [35].

Since microorganisms with inulinase activity rarely produce 2,3-BD, the conversion of inulin to 2,3-BD in a one-step SSF is usually achieved via the simultaneous hydrolysis of the substrate along with fermentation via pure enzyme addition. There are only a few examples of strains that combine hydrolytic with producing properties and are capable of the direct synthesis of 2,3-BD from inulin: the *Paenibacillus polymyxa* strains ZJ-9 and ATCC 12321 [22,36], *Bacillus* sp. [20], and *K. pneumoniae* H3 [34]. In all these cases, however, the inulin used as a substrate is short-chained or hydrolyzed. The highest concentrations of 2,3-BD from inulin so far have been obtained by Gram-negative bacteria: *K. pneumoniae* CICC 10011 (91.6 g/L) and *K. pneumoniae* H3 (80.4 g/L) from Jerusalem artichoke powder. However, to increase the sugar amount, the first team added inulinase with a dosage of 2 U/g of substrate [21], while in the second study, acid hydrolysis (at pH 3.0) was applied [34].

The 2,3-BD titer of 67.5 g/L reached by *B. licheniformis* 24 from Frutafit^®^ CLR inulin is impressive, although the strain was not able to hydrolyze the raw Frutafit^®^ HD. The profiles of the inulin substrates used show that the content of mono- and disaccharides, as well as the DP of the inulin molecules, significantly influence the amounts of fermentation products obtained. The complete consumption of glucose, fructose, and sucrose after the first eight hours of fermentation obviously triggered the overexpression of the studied genes, all of them reaching peak values in the mRNA level after 24 h (Table 2).

The obtained results show that the key gene for Inulin hydrolysis is *sacC*, encoding fructan β-fructosidase (levanase) of the GH32 family. It shows vast upregulation at pH 6.25; at the same time, the second greatly upregulated gene, *sacB*, is unable to degrade inulin [37]. *SacB* gene overexpression coincided with an obvious increase in the viscosity of the culture medium, suggesting EPS synthesis.

**Figure 6:**
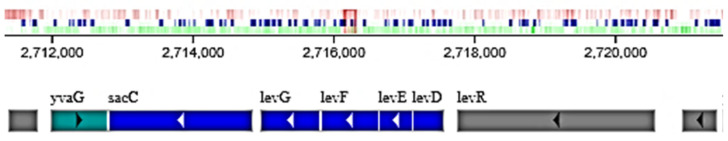
Schematic presentation of *B. licheniformis* ATCC 14580^T^ chromosome taken from KEGG database (https://www.genome.jp/genome/bli+BL03120, accessed on 1 August 2023). Designation of the genes and the enzymes encoded by them: *levR*, transcriptional regulator; *levD*, fructose PTS system EIIA component; *levE*, fructose PTS system EIIB component; *levF*, fructose PTS system EIIC component; *levG*, fructose PTS system EIID component; and *sacC*, fructan β-fructosidase.

This observation is in agreement with Li et al. [24], the first researchers who suggested the involvement of SacC in inulin hydrolysis by *B. licheniformis*. These authors reported that the specific activity of the purified enzyme was 987.0 U/mg, ten times higher than that of *B. subtilis* levanase [38], and at a pH optimum of 6.5. Later, Park et al. [20], after the estimation of the Km and Vmax of the purified enzyme, also supported this statement, revealing that the highest activity of SacC was obtained with levan (100%), followed by inulin (87.6%), and sucrose (12.5%). Figure 6 shows the surroundings of *sacC* in the *B. licheniformis* genome. *SacC* is clustered in an operon structure following *levD, levE, levF,* and *levG*, which encode proteins of the sugar-transporting phosphotransferase system (PTS).

The first level of control of the levanase operon is the probable global regulation (catabolite repression by glucose), while the second (supported by our experiments) includes activation by fructose [39] and the participation of the positive regulator LevR [40]. In *B. subtilis*, in the presence of glucose, the phosphorylated CcpA repressor binds the *cis*-acting catabolite-responsive element (*CRE*) preventing the transcription of the levanase operon [41]. However, the *CRE* site with sequence TGAAAACGCTT(a)ACA proposed by Marciniak et al. [39] is not located upstream of *sacC* in the genome of the strain *B. licheniformis* 24 and ATCC 14580^T^; therefore, this mechanism most likely does not operate in this species. Thus, *sacC* expression control in *B. licheniformis* is most probably governed by LevR as an operon activator and by LevE, which is known to control *levR* activity [42].

**Figure 7:**
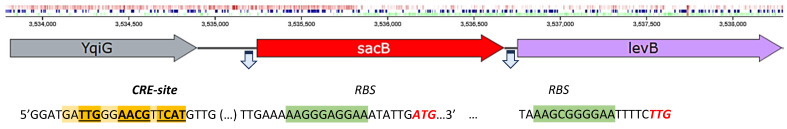
Genomic context of *sacB* in the chromosome of *B. licheniformis* ATCC 14580^T^. Designation of the genes and the enzymes encoded by them: *sacB*, levansucrase; *levB*, levanase; and *yqiG*, NADH-dependent flavin oxidoreductase. *Cre* site sequence upstream *sacB* is boxed, and the mandatory nucleotides are shown in orange; the non-mandatory nucleotides, which are a part of the *cre* site, are shown in yellow. RBS (Shine–Dalgarno) sequences upstream of *sacB* and *levB* are highlighted in green; the start codons are red.

Another highly upregulated gene, *sacB* encodes levansucrase (2.4.1.10). This enzyme belongs to the group of non-Leloir glycosyltransferases, often called sucrases because they hydrolyze sucrose first, and then use the glucose (glucosyltransferases) or the fructose (fructosyltransferases) for the synthesis of various oligosaccharides. The intermediate glycosyl-enzyme complex can further be hydrolyzed or, in the presence of a suitable acceptor, donate its glycosyl group and form a di- or oligosaccharide [43,44]. The transfructosylation activity of levansucrase is much better studied than its hydrolytic ability, which is generally regarded as an undesirable effect in the synthesis of fructooligosaccharides (FOS) with presumably prebiotic properties. Considerable invertase activity has been reported in various bacteria, for instance, *B. circulans* [45]. In general, both hydrolase and transglycosidase activities obey Michaelis–Menten kinetics, but the balance between them is delicate and depends on numerous factors, such as pH [46]. Levansucrases are widely distributed across bacterial genera, including *Bacillus*, but reports in *B. licheniformis* are relatively scarce [42,47]. In *B. licheniformis* RN-01, a purified 52 kDa protein is able to synthesize levan with different molecular weights at different temperatures [48]. Levansucrase from *B. licheniformis* 8-37-0-1, a 51 kDa monomer similar to that of *B. licheniformis* 24 (Table 1), was heterologously expressed in *E. coli* and showed broad trans-fructosylation activity. Using sucrose as a donor, the enzyme was able to transfer a fructosyl moiety to galactose, cellobiose, xylose, maltose, lactose, arabinose, and trehalose, as well as synthesize notable quantities of levan [49]. *B. licheniformis* ANT 179, isolated from Antarctic soil, was shown to possess an extracellular levansucrase and produce levan and inulin-type FOS [50]. The genomic context of *sacB* in the chromosome of *B. licheniformis* ATCC 14580^T^ is presented in Figure 7.

A typical *CRE* site (containing all mandatory motifs) was found in the sequence upstream of the *sacB* gene in *B. licheniformis* (Figure 7). In contrast with *B. subtilis*, in which *sacB*, *levB* (*yveB*), and *yveA* are organized in a tricistronic operon [51], *B. licheniformis* lacks a *yveA* analog. In the ATCC 14580^T^ chromosome, next to *levB* is located a gene encoding a transfer RNA (tRNA-Arg), followed by other genes likewise unrelated to *sacB*.

Notably, our RT-PCR experiments showed independent expressions of *sacB* and *levB*. Under fermentation conditions of pH 6.25, *sacB* was 196.72 times overexpressed, while *levB* remained unaffected, thus suggesting that the non-coding region (76 bp) between the genes probably prevents their common expression control. Regarding inulinase activity, it is known that levansucrase can break β-2,6 linkages, stopping hydrolysis when reaching a β-2,1 linkage [37,42], thus being rather unrelated to the degradation of inulin. The main effect of the overexpression of *sacB* is the production of EPS at pH values above 5.75, giving high visual viscosity to the culture liquid, increasing at pH 6.25, corresponding to *sacB* gene overexpression. Unfortunately, due to residual inulin in the fermentation medium, the quantity of EPS cannot be precisely determined due to the impossibility of separate extraction.

However, it is clear that part of the carbon from the substrate was lost because it was converted to EPS by *B. licheniformis* 24. Considering sucrose utilization, levansucrase exhibits impressive sucrase activity. SacB in *B. licheniformis* TKU004 (with pomelo albedo powder as a carbon source) hydrolyzes the tri-saccharide raffinose (gal-glu-fru) and the tetrasaccharide stachyose (gal-gal-glu-fru) by attacking the glycoside bond between the glucosyl and fructosyl moieties. In stark contrast, FOS were hydrolyzed more than five times (19%) less strongly than sucrose [28], supporting the conclusion that the main substrate of *SacB* in the processes carried out was sucrose.

LevB, according to Jensen et al. [52], is also not able to hydrolyze the β-1,2-linked units in inulin. However, the simultaneous action of the enzymes encoded by the *sacB* and *levB* genes leads to the release of fructose molecules, and the action of *levB* as an endo-levanase stimulates the activity of *sacB* as an exo-levanase [53].

**Figure 8:**
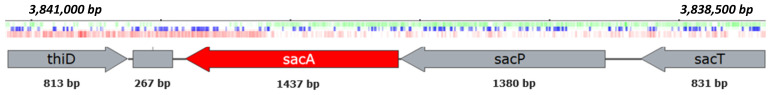
The gene *sacA* and its regulatory region in the *B. licheniformis* ATCC 14580^T^ chromosome. Designation of the genes and the enzymes encoded by them: *sacA*, β-fructofuranosidase; *sacP*, sucrose PTS system EIIBCA (or EIIBC) component; *sacT*, β-glucoside operon transcriptional anti-terminator (BglG family); 267 bp, encoding a hypothetical protein (with ID QCY01196.1); and *thiD*, pyridoxal kinase.

The genes *sacP* and *sacA* are organized in an operon with a common sucrose-inducible promoter that is activated in a medium containing sucrose [54]. The *sacP* gene encodes a component (EIIBCA or EIIBC) of a phosphoenolpyruvate-dependent, sugar-transporting phosphotransferase system (PEP-PTS), which transports sucrose from the cell periplasm into the cytoplasm and simultaneously phosphorylates it [55]. Sucrose 6-phosphate is then hydrolyzed by the endocellular hydrolase SacA to fructose and glucose 6-phosphate. The regulation of *sacA* expression is driven by *sacP* at positions 3,839,940 to 3,841,319, upstream of *sacA* in *B. licheniformis* ATCC 14580^T^ chromosome (Figure 8).

In *B. subtilis*, the *sacP* gene is located within the *sacPA-ywdA* operon [56]. The *B. licheniformis* ATCC 14580^T^ chromosome lacks *yweA*, as the partial sequence of 267 bp adjacent to *sacA* does not possess similarity to this gene. However, most probably, the operon is positively regulated by the anti-terminator encoded by *sacT*.

**Figure 9:**
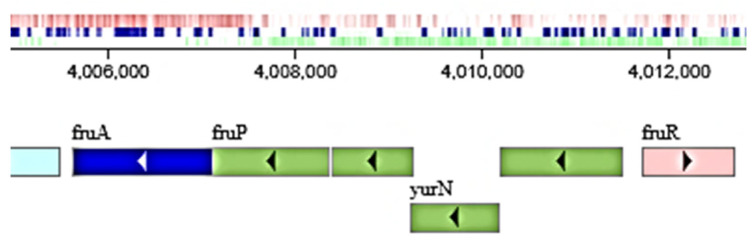
Genomic context of the *fruP* and *fruA* genes in the *B. licheniformis* ATCC 14580^T^ chromosome as presented in the KEGG database (https://www.genome.jp/genome/bli+BL03120, accessed on 18 August 2023).

Interestingly, the *B. licheniformis* genome contains an additional β-fructosidase operon, *fruP-fruA*, encoding an enzyme of the GH32 family (EC 3.2.1.26) and the MFS (major facilitator protein) transporter. This operon is preceded by three genes encoding fructooligosaccharide transport system substrate-binding proteins (green in Figure 9).

The operon is probably regulated by *fruR*, encoding the LacI family transcriptional regulator of 324 amino acids (NCBI ID AAU25617). Since *fruA* is significantly upregulated, the elucidation of its involvement in inulin hydrolysis may deserve future attention.

## 4. Materials and Methods

### 4.1. Strain, Media, and Cultivation Conditions

*B. licheniformis* 24 was previously isolated from a soil sample in Bulgaria [11], identified using 16S rRNA gene sequencing (GenBank accession no. MK461938.1), and stored in the microbial collection of the Institute of Microbiology, the Bulgarian Academy of Sciences.

The strain was maintained in slant LB agar tubes at 4 °C (Alfa Aesar GmbH & Co. KG; Karlsruhe, Germany) or as a frozen liquid culture supplemented with 20% glycerol at −70 °C.

As a substrate in all fermentation processes, the soluble chicory flour Frutafit^®^ CLR (Sensus B.V., Roosendaal, The Netherlands) was used.

The flask-batch cultivation of *B. licheniformis* 24 was carried out in 500 mL flasks containing 100 mL of medium, with previously optimized content [14], and 50 g/L of the chicory flour Frutafit^®^ CLR substrate instead of glucose. As the inoculum (1%), an overnight culture with OD_600_ = 2.0 was used. The flasks were incubated in a rotary shaker at 37 °C and 200 rpm.

Batch processes with pH and aeration control were performed in a 1 L stirred bio-reactor (Biostat^®^ A Plus, Sartorius Stedim Biotech, Gottingen, Germany), equipped with an air pump and rotameter. The same optimized medium was used [14] as the substrate was 200 g/L of the chicory flour Frutafit^®^ CLR instead of glucose. The process parameters were maintained at their optimal values: temperature of 37.8 °C and aeration of 3.68 vvm [14,16]. pH was maintained with the addition of 6M of NaOH and 5M of HCl. The inoculum was 10% (overnight culture of *B. licheniformis* 24 with OD_600_ = 2.0).

### 4.2. DNA Isolation, PCR, and Sequencing

Total DNA and RNA from samples taken at different hours were isolated with the GeneMATRIX Bacterial & Yeast Genomic DNA Purification Kit and the GeneMATRIX Universal Purification Kit, respectively, according to the instructions of the manufacturer (EURx, Gdansk, Poland).

PCR was performed with TaKaRa Taq Version 2.0 (Clontech Laboratories, Inc., A Takara Bio Company, Mountain View, CA, USA) in a 25 μL reaction volume with 50 ng of the DNA template and 0.4 μM of the primers (Table 3 using a QB-96 Satellite Gradient Thermal Cycler (LKB Vertriebs GmbH, Vienna, Austria).

Genes responsible for inulin hydrolysis were amplified for 35 cycles of denaturation, annealing, and elongation (10 s at 98 °C, 40 s at 63 °C, and 2 min at 72 °C, respectively). Initial denaturation was set at 94 °C for 3 min and final elongation at 72 °C for 5 min. The optimal temperature of annealing was determined with gradient analysis. The gene for 16S rRNA was amplified with the universal primers 27F and 1492R for 35 cycles of denaturation (94 °C, 1 min), annealing (58 °C, 45 s), and elongation (72 °C, 2 min), initial denaturation at 94 °C for 3.30 min, and final elongation at 72 °C for 7 min.

PCR products were visualized on 1% agarose gel with SimplySafe (EURx, Gdansk, Poland) and sent for sequencing to Macrogen Inc. (Amsterdam, The Netherlands).

The obtained sequences were analyzed. The low-quality parts were trimmed using Chromas Lite version 2.1 https://chromas-lite.software.informer.com/2.1/ (accessed on 10 June 2023), assembled employing Cap3 software (https://doua.prabi.fr/software/cap3) (accessed on 3 August 2023), and compared with the NCBI database using BLASTN and BLASTX. The deduced amino acid sequences were obtained using the free software Expasy Translate Tool version 2 (SIB Swiss Institute of Bioinformatics) (https://web.expasy.org/translate/) (accessed on 3 August 2023). The sequences deriving from the genome of *B. licheniformis* strain 24 were deposited in NCBI GenBank with the accession numbers as follows: *sacA*, OR400366; *sacB*, OR400367; *sacC*, OR400368; *levB*, OR400369; and *fruA*, OR400370.

### 4.3. Real-Time-RT PCR

Reverse transcription was performed with the NG dART RT Mix (EURx, Gdansk, Poland) in 20 μL reactions with 1 μg of total RNA, 200 ng of random hexamer primers, and the following program: 10 min at 25 °C for primer hybridization, 50 min at 50 °C for the reverse transcription itself, and 5 min at 85 °C for the inactivation of the enzyme. Prior to reverse transcription, the RNA samples were treated with 5U dNase I in a buffer with 25 mM of MgCl_2_ (EURx, Gdansk, Poland) for 30 min at 37 °C. The enzyme was inactivated for 10 min at 65 °C in the presence of 20 mM of EDTA.

Real-time PCR was performed with a SsoFast™ EvaGreen^®^ Supermix with Low ROX (Bio-Rad, Hercules, CA, USA) in a Corbett Research RG-6000 Real-Time PCR Thermocycler (Qiagen, Germantown, MD, USA). Primer pairs were generated using Primer-BLAST [57] and targeted fragments from 62 to 125 bp within five genes involved in inulin hydrolysis (Table 4).

The optimal annealing temperature was determined to be 65 °C. Each reaction of 20 μl contained 40 ng of cDNA as a template and 500 nM of the primers. 16S rRNA was used as an internal control for each sample in each run. The beginning (0 h) at the lowest pH (5.2) was used as a basis for comparison. Relative expression was calculated using the ΔΔCt method as follows:

ΔCt = Ct_gene_ − Ct_16S_

ΔΔCt = ΔCt_pH5.5/5.7/6.0/6.2_ − ΔCt_pH5.2/0h_

Fold Change (FC) = 2^−ΔΔCt^

The values thus obtained mean the following: 1.00—no change in expression; 2.00—twofold higher expression; and 0.50—two-times lower expression.

### 4.4. Analytical Methods

The cell growth was monitored via the measurement of the optical density (OD) at a wavelength of 600 using a UV/VIS spectrophotometer (Thermo Scientific Inc., Waltham, MA, USA). RNA concentrations and purity (Abs_260_/Abs_280_ ratio) were determined using a Quawell UV Spectrophotometer Q3000 (Quawell Technology, Inc., San Jose, CA, USA).

The sugar content in the substrate was analyzed with a YL Instrument 9300 HPLC System (YL Instrument Co., Ltd., Anyang, Republic of Korea). An HPLC column Aminex HPX-87H at 65 °C with a mobile phase of 5 mM of H_2_SO_4_ at a flow rate of 0.6 mL/min (BioRad Laboratories, Hercules, CA, USA) was used for 2,3-BD, acetoin, and sugars quantification. Glucose, fructose, sucrose, and oligosaccharides contained in pure chicory flour powders were separated with an HP-Amino (5 µm, 120 Å, 4.6 × 250 mm) column (Sepax Technologies, Inc., Newark, DE, USA) at 25 °C and detected with an RI detector (YL 9170) at 35 °C. As a mobile phase, a mix of water and acetonitrile at a ratio of 30:70 (*v*/*v*) with a flow rate of 1.0 mL/min was used.

## 5. Conclusions

The present paper sheds light on the complex nature of substrate hydrolysis in the course of 2,3-butanediol production from inulin by *B. licheniformis*. Our results reveal that the composition and oligosaccharide structure of the inulin-containing substrates is of great importance for the yield of the target metabolite. Thus, via the fermentation of soluble inulin from the chicory flour Frutafit^®^ CLR at 67.5 g/L, 2,3-BD was produced. To our knowledge, this concentration is the highest obtained by non-engineered a *B. licheniformis* strain and without prior substrate hydrolysis. Of the five investigated genes potentially involved in inulin hydrolysis, four were overexpressed under the optimal conditions for 2,3-BD synthesis at pH 6.25 (*sacC*, *sacA*, *fruA*, and *sacB*), while the *levB* gene expression level remained unchanged. After analyzing the structures of the encoded enzymes and the genomic context, it can be concluded that SacC, SacA, and FruA have roles in the hydrolysis of inulin, while the overexpression of *sacB* has no positive effect on inulin degradation. SacB is related mostly to EPS production, which channels part of the carbon in the substrate in a disadvantageous direction. Therefore, future inulin-based microbial production of 2,3-BD by *B. licheniformis* should lead to the development of improved strains with the *sacB* gene knocked out.

## Figures and Tables

**Table 1:** Genes encoding carbohydrate-active enzymes involved in inulin hydrolysis by *B. licheniformis* 24.

Gene	Size (bp)	Enzyme (EC Number)	Enzyme (Name)	Localization	Amino Acids (Number)	CAZy (Family)	GenBank Accession No.
*sacA*	1437	3.2.1.26	β-Fructofuranosidase	Cytoplasmic	479	32	OR400366
*sacB*	1449	2.4.1.10	Levansucrase	Extracellular	482	68	OR400367
*sacC*	2034	3.2.1.80	Fructan β-fructosidase	Extracellular	677	32	OR400368
*levB*	1548	3.2.1.65	Levanase	Membrane	516	32	OR400369
*fruA*	1479	3.2.1.26	β-Fructofuranosidase	Cytoplasmic	492	32	OR400370

**Table 2:** Fold changes (FCs) in gene expressions of genes involved in inulin hydrolysis during processes with pH maintained at the values indicated. Fold change was calculated vs. 0 h at pH 5.25. The relative abundance of each gene in the mRNA level was estimated according to the comparative ΔΔCt method. Expression was normalized to the 16S rRNA gene as an endogenous control. FC 1.00 indicates no change in expression; FC < 1.00 indicates decreased expression (e.g., 0.5 means twice-lower expression); FC > 1.00 indicates increased expression (e.g., 2 means twice-higher expression); ND = not detected. The presented results are mean values of two independent fermentation experiments, each tested with three independent RT-PCR trials. The standard deviation was below 5%.

Gene	Time (h)	FC				
		pH 5.25	pH 5.50	pH 5.75	pH 6.00	pH 6.25
*sacA*	0	1.00	0.20	0.06	0.61	0.29
	24	0.25	0.88	10.34	ND	66.26
	48	0.41	0.97	0.58	3.32	1.32
	72	1.00	1.02	9.51	1.87	1.08
*sacB*	0	1.00	0.91	1.34	1.41	1.17
	24	0.74	1.39	12.82	ND	196.72
	48	0.67	1.99	0.37	1.85	1.58
	72	0.29	1.73	7.16	5.31	0.13
*sacC*	0	1.00	0.20	0.80	0.43	0.49
	24	0.85	1.19	23.59	22.63	163.14
	48	1.11	2.62	3.89	3.56	1.21
	72	2.11	ND	1.96	1.31	0.22
*levB*	0	1.00	0.30	0.57	0.11	0.33
	24	0.41	2.75	1.02	1.04	0.40
	48	0.36	1.53	0.56	1.06	0.74
	72	0.61	2.58	ND	0.86	0.84
*fruA*	0	1.00	0.11	0.13	0.52	0.63
	24	0.90	1.17	8.06	2.91	53.82
	48	0.43	1.16	0.56	7.67	3.46
	72	2.50	4.14	5.10	8.40	0.42

**Table 3:** Nucleotide sequences of the primers used for gene amplification and sequencing.

Primer	Sequence (5′-3′)	PCR Product (bp)	Position in Genome *
sacA_F	atgaatcaagatcaggagcttcgtcaaaaggcaat	1437	3,838,486–3,839,922
sacA_R	ttatgccattgtccaggatgtcacattcattatga		
sacB_F	atgaacatcaaaaacattgctaaaaaagcgtcagc	1449	3,535,232–3,536,680
sacB_R	ttatttgtttaccgttagttgtccctgttcaagga		
sacC_F	cgctgcctggatgcttcgcaaaggggtgaatcc	2556 ^†^	2,712,789–2,714,822
sacC_R	gaccgtcaatacggttatgccgggctcaacc		
levB_F	ttgaagaaggcagtatataagcggatcagcatttt	1548	3,536,757–3,538,304
levB_R	taatcgcggattgaacgcaaatgtttgatcttaaga		
fruA_F	atgaacagaattcagcaggcagaagaagcattaaa	1479	4,005,611–4,007,089
fruA_R	tcatttggcttcatcacctttccaaatatctttca		

* Positions are according to the genome of *B. licheniformis* ATCC 14580 (CP034569.1). ^†^ Primers sacC_F/sacC_R target 220 bp downstream and 302 bp upstream of the gene *sacC*.

**Table 4:** Primers used in real-time PCR experiments.

Primer	Sequence (5’-3’)	PCR Product (bp)	Position in Gene *
16S_F	gagtacgaccgcaaggttga	100	875–895
16S_R	cctggtaaggttcttcgcgt		975–955
sacA_RTF	aagagatcgccctcacgccgagcgactggttt	125	255–286
sacA_RTR	atttccctcgccgtctctgacattccccgtgt		379–348
sacB_RTF	caacagagcctactacgggggcagcaagaagt	117	861–892
sacB_RTR	tcgatgattccgagagcgccgttagccagcga		977–946
sacC_RTF	gccgctcgttgccatttatacgcaggaccgga	64	375–406
sacC_RTR	gctgtaggcgatgctttgcacttgttccccgc		438–407
levB_RTF	gcatactggacaggcagcttcaacggcaacga	121	784–815
levB_RTR	cgttcgtttcgccgtcctcaaatgtcacgccc		904–873
fruA_RTF	gggagtcagagatgccgacgaaagcagacgga	62	893–924
fruA_RTR	ttcacgcggcaaagttaatgccccgcaccatc		954–923

* Positions are according to the respective gene sequences of *B. licheniformis* ATCC 14580^T^ (NCBI GenBank acc. no. CP034569.1).

## Data Availability

The nucleotide sequences are available in NCBI GenBank. The strain *B. licheniformis* 24 can be obtained from the authors upon request.
